# Patient Perceptions and Acceptance of Blockchain-Based Health Data Sharing in Oncology: Cross-Sectional Survey

**DOI:** 10.2196/89278

**Published:** 2026-06-25

**Authors:** Matheus Villa Moraes, Marcos Almeida Matos

**Affiliations:** 1 Department of Oncology, Departament of Health Technologies, Hospital Santa Izabel, Escola Bahiana de Medicina e Saúde Pública, Praça Conselheiro Almeida Couto, 500 Nazaré, Salvador, Bahia, Brazil, 55 71991929771

**Keywords:** blockchain, health data sharing, oncology, patient perceptions, digital health, data governance, cross-sectional survey, Brazil

## Abstract

**Background:**

Fragmentation of electronic health records in oncology hinders coordinated care, delays diagnoses, and limits therapeutic personalization. Blockchains promise to promote secure, interoperable, and patient-centered data governance; however, patient perceptions of blockchains remain underexplored, particularly in middle-income countries such as Brazil.

**Objective:**

We assessed opinions, attitudes, and willingness among patients with cancer to digitally share clinical information and the feasibility of applying blockchains to restructuring secure health data sharing in the Brazilian public health context. We had three research questions: (1) What is the level of digital health tool acceptance among patients with cancer in Brazil? (2) Which sociodemographic factors are associated with willingness to share health data? (3) Are blockchains feasible and acceptable for restructuring secure oncology data sharing?

**Methods:**

An exploratory, descriptive, cross-sectional self-report survey was conducted at Hospital Santa Izabel, a national oncology reference center in Salvador, Bahia, Brazil, between September and November 2023. A convenience sample of 110 outpatients with cancer was recruited systematically; data were collected via a self-administered questionnaire. The 20-item instrument, developed de novo and validated via expert panel and pilot testing, covered 5 content domains yielding 3 composite scoring domains: self-management, adherence, and governance. We used Cronbach α to assess internal consistency, independent 2-tailed *t* tests, 1-way ANOVA, and Pearson correlations to compare domain scores across sociodemographic groups, and a chi-square goodness-of-fit test to examine trust proportions across recipient types.

**Results:**

We received sufficiently complete responses from 94.5% (104/110) of patients. The sample was predominantly female (63/98, 64.3%), self-identified as *pardo* (mixed-race; 64/98, 65.3%), and lower income (55/96, 57.3% earned less than twice the minimum wage). Acceptance of technology was high: 86.4% (95/110) would use health apps and 89.1% (98/110) expressed interest in prevention-focused applications. Trust in data sharing varied significantly across recipient types (*χ*^2^_3_=210.4; *P*<.001): 79.1% (87/110) trusted health care professionals, 51.8% (57/110) hospitals, 15.5% (17/110) the pharmaceutical industry, and 10% (11/110) the government. Anonymization and encryption significantly increased willingness to share (92/110, 83.6%). Younger patients (18‐59 years) showed significantly higher adherence scores than those aged ≥60 years (mean 76.49, SD 19.16 vs mean 65.83, SD 26.66; *t*_95_=2.29; *P*=.02). Domain reliability was good to excellent (Cronbach α=0.8807 [adherence], 0.8504 [self-management], and 0.7576 [governance]).

**Conclusions:**

Patients with cancer in Brazil demonstrated high acceptance of digital health tools and openness to data sharing when privacy, security, and governance are guaranteed. This supports the feasibility of blockchain-based health data management systems, provided they incorporate patient-centered principles, digital inclusion strategies, and robust governance aligned with Brazilian regulations (the General Data Protection Law) and the Unified Health System (Sistema Único de Saúde) infrastructure. Importantly, patient support reflected acceptance of blockchain’s functional principles, data security, anonymization, and auditability rather than familiarity with the technology itself, a distinction with direct implications for future implementation studies.

## Introduction

The complexity of oncology treatment requires efficient coordination among multidisciplinary teams, integrating clinical data, examinations, medical history, and therapies. However, the fragmentation of electronic health record (EHR) systems hinders this integration, causing delays in diagnosis, failures in continuous monitoring, and limitations in therapeutic personalization—factors that negatively impact clinical outcomes [1,2].

Given the rapid evolution of digital health, a coordinated and evidence-based approach is essential for technological innovations to effectively meet the needs of health systems [3,4]. The World Health Organization recognizes the potential of digital technologies to support the Sustainable Development Goals and universal health coverage, fostering initiatives that discuss governance and the ethical use of health data [5]. However, public acceptance regarding the use and sharing of personal information remains limited. Studies show that individuals have concerns about privacy and security and tend to better accept data sharing when there is transparency, informed consent, and personal control [6].

Recent evidence highlights that patient attitudes toward data sharing are shaped by trust in health care institutions, perceived utility of digital tools, and concerns about secondary use of information, including by artificial intelligence systems [7-10]. These factors are particularly relevant in oncology, where patients navigate heightened emotional vulnerability and complex therapeutic trajectories that generate large volumes of sensitive clinical data [11-13].

Interoperability between health systems is essential, as it concerns the ability of different technologies and institutions to access, exchange, and use data in a coordinated, secure, and effective manner [14]. Resources such as wearable sensors, applications, and intelligent algorithms have benefited both clinical practice and oncology studies by allowing continuous, multisource data collection [15-17].

Digital transformation has also impacted the Brazilian public sector, with the Unified Health System (Sistema Único de Saúde [SUS]) progressively incorporating remote monitoring and integrated data management, especially relevant in oncology [18]. The National Health Data Network (Rede Nacional de Dados em Saúde [RNDS]), linked to the Conecte SUS program, operationalizes Brazil’s Digital Health Strategy through standardization, security, and structured sharing of clinical data [19,20].

From this perspective, blockchain technology emerges as a favorable alternative to address historical challenges related to fragmentation, security, traceability, and governance of health information. Its use enables the creation of immutable, decentralized, and auditable records, granting patients greater control over their clinical data and promoting interoperability between institutions and EHR systems [21-25]. Blockchain supports automated informed consent, granular access management, and real-time auditable documentation of clinical interactions. In clinical research, blockchain strengthens data integrity and reduces risks of manipulation, increasing scientific reliability [26-28].

In practical terms, a blockchain-based EHR system in oncology would function as follows: each patient’s clinical data—including imaging results, molecular profiles, therapeutic history, and follow-up records—would be encrypted and stored across a distributed network of authorized nodes. Patients would hold cryptographic keys granting them granular control over who accesses their data and for what purpose. Smart contracts would automate informed consent processes, ensuring that data are only shared under prespecified, auditable conditions. Health care institutions and research entities would access data via a permissioned blockchain network, with every access event permanently logged and visible to the patient. This architecture, demonstrated in prototypes such as the Massachusetts Institute of Technology’s MedRec system [29] and evaluated in Chinese health informatics contexts [30], operationalizes transparency and patient-centered governance at the technical level.

Despite its potential, large-scale adoption requires overcoming regulatory, technological, and cultural barriers, as well as integration with legacy systems and compliance with international data protection standards. Blockchain must be implemented alongside advanced cryptographic techniques, decentralized digital identity strategies, and robust participatory governance models [31-33]. The formulation of effective digital health solutions requires approaches centered on equity, interdisciplinary collaboration, and responsible governance [34,35].

Understanding patient perceptions is critical before implementing any blockchain-based system. Active patient participation can stimulate greater acceptance, adherence, and confidence in digital solutions, contributing to more ethical and socially responsible data governance [36,37].

**Figure 1.**
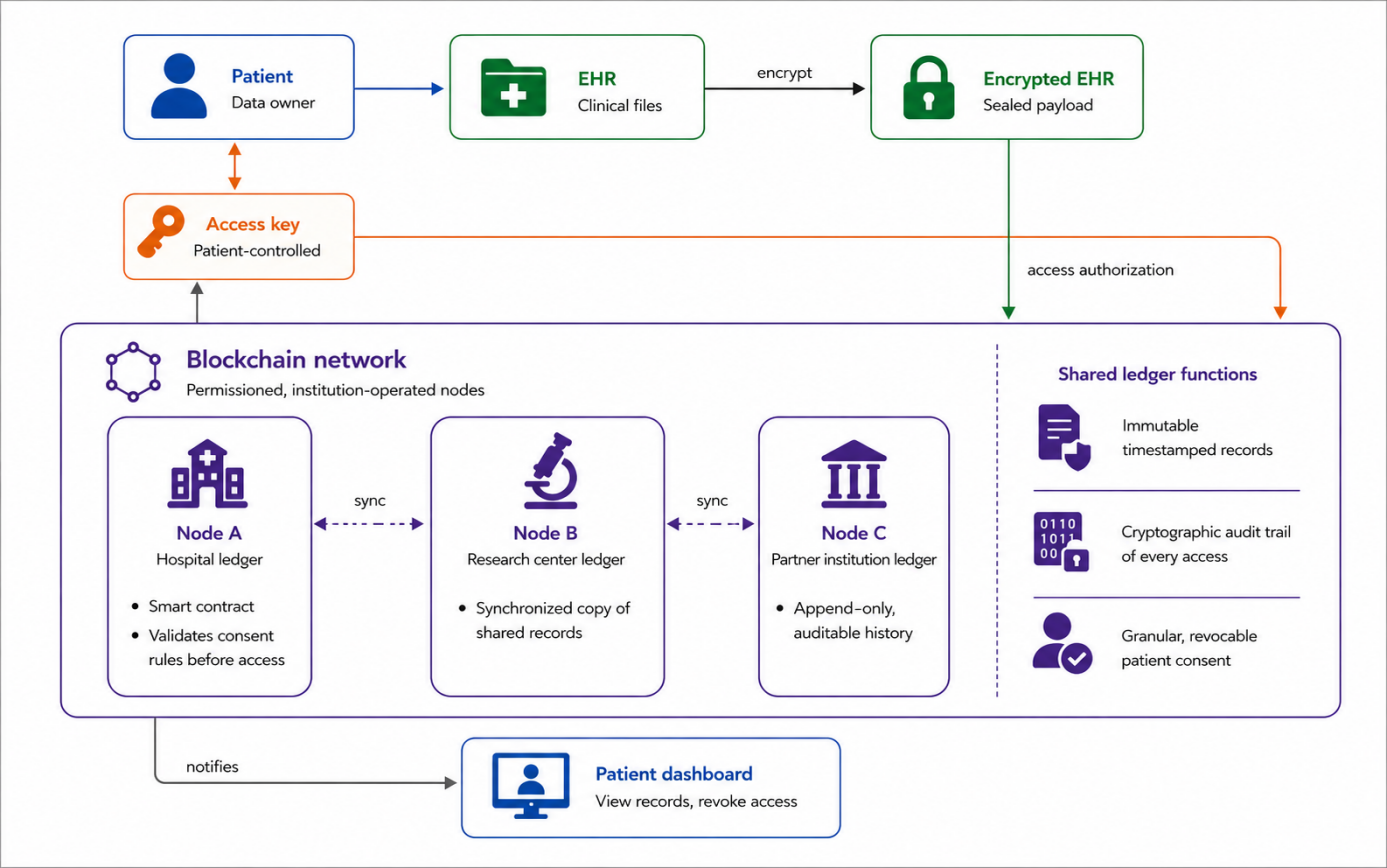
Conceptual architecture of a blockchain-based electronic health record (EHR) system. Patients control access to encrypted medical records via cryptographic keys. Clinical data are shared among authorized health care institutions and research entities through a permissioned blockchain network. The model emphasizes data security, patient-centered access governance, and institutional interoperability. Adapted from Leoratto [38].

This study therefore aimed to investigate the opinions and attitudes of patients with cancer regarding the sharing of clinical information, evaluating the feasibility and acceptance of a blockchain-based approach to restructure models of continuous and secure oncology data sharing. Three research questions guided the study: (1) What is the level of acceptance of digital health tools among patients with cancer in Brazil? (2) What sociodemographic factors are associated with willingness to share health data? (3) Is a blockchain-based approach considered feasible and acceptable as a framework for restructuring secure oncology data sharing within the Brazilian SUS context? Figure 1 illustrates the proposed blockchain-based framework for secure electronic health record (EHR) management and sharing, highlighting patient-controlled access authorization, data encryption, and interoperability among health care and research institutions.

## Methods

### Ethical Considerations

This study was conducted in accordance with the ethical principles outlined in the Declaration of Helsinki and complied with all applicable national regulatory standards. The research protocol was reviewed and approved by the institutional review board of Hospital Santa Izabel, under the Certificate of Presentation for Ethical Consideration number 70726523.3.0000.5520, with approval opinion number 068562/2023 (Multimedia Appendix 1). All participants provided digital informed consent prior to inclusion. Consent was documented through electronic confirmation via REDCap (Research Electronic Data Capture). All procedures adhered to the Brazilian General Data Protection Law (Lei Geral de Proteção de Dados [LGPD]), guaranteeing anonymity, secure data handling, and restricted access to collected information.

### Study Design

This exploratory and descriptive study adopted a quantitative cross-sectional self-report survey design to evaluate patients with cancer’ perceptions regarding health data sharing, self-management, and trust in digital health solutions, considering the potential feasibility of blockchain as an enabling technology [36,37]. This study followed the STROBE (Strengthening the Reporting of Observational Studies in Epidemiology) reporting guidelines for cross-sectional studies. A completed STROBE checklist is provided as Checklist 1.

### Study Setting and Recruitment

The study was conducted at Hospital Santa Izabel, located in Salvador, Bahia, Brazil, a national oncology reference center integrated within the ecosystem of scientific, technological, and innovation institutions. Data collection occurred between September and November 2023.

Recruitment followed a systematic convenience approach. Clinical staff—nurses and attending oncologists—identified eligible patients during outpatient appointments in both SUS and private insurance oncology clinics. Eligible patients were personally invited by a trained research team member who provided standardized verbal information about the study objectives, voluntary participation, and confidentiality. Those who agreed to participate were provided access to the questionnaire via a QR code displayed in waiting areas and consulting rooms. The questionnaire was self-administered on participants’ own personal devices or on an institution-provided tablet when needed, without direct researcher intervention during completion, ensuring participant autonomy and spontaneous responses. Participation had no influence on clinical care or the patient–health care provider relationship.

### Participants

A convenience sample of 110 patients with cancer undergoing treatment or follow-up at Hospital Santa Izabel was included. Given the exploratory nature of the study and the absence of a primary hypothesis requiring formal power calculation, sample size was determined based on institutional feasibility and methodological guidance. A sample of n≥100 is sufficient to detect medium-sized effects (Cohen *f*=0.25) with approximately 80% power in ANOVA comparisons between 2 and 4 groups and to ensure sufficient diversity of sociodemographic profiles for descriptive and comparative analyses [39].

The inclusion criteria were as follows: adults aged 18 years or older; patients undergoing active oncology treatment or follow-up; and patients with an Eastern Cooperative Oncology Group (ECOG) performance status of 0, 1, or 2.

The exclusion criteria were as follows: patients with advanced or terminal disease (ECOG performance status 3 or 4) and patients who did not provide responses to the items comprising the 3 composite scoring domains (self-management, adherence, and governance).

### Questionnaire Development and Validation

The development and validation of the data collection instrument followed a structured multistep process.

#### Literature Review

A systematic literature search was conducted in the PubMed, Scopus, Web of Science, and SciELO databases, combining Boolean operators with terms including *blockchain* AND *health data*, *digital health* AND *patient perception*, *data sharing* AND *oncology*, and *privacy* AND *health information*. Articles published within the last 10 years addressing digital health technologies, health information security, and data governance were considered.

#### Instrument Development

All 20 questionnaire items were developed de novo by the research team, as no validated instrument addressing blockchain acceptance in the Brazilian oncology context was identified. The questionnaire was developed and administered entirely in Brazilian Portuguese. All items used closed-ended response formats (single-choice or multiple-choice checkboxes); no open-ended questions were included. Importantly, the questionnaire did not ask patients to evaluate “blockchain” as a named technology; rather, items assessed attitudes toward the functional properties that blockchain enables—data security, anonymization, encryption, patient-controlled access, auditability, and institutional transparency. Accordingly, the instrument captured acceptance of these functional principles rather than informed endorsement of blockchain as a specific technology. The semistructured questionnaire (Multimedia Appendix 2) was organized into five content domains: (1) sociodemographic characteristics, (2) familiarity with and perception of emerging technologies, (3) data security and privacy, (4) willingness to share medical information, and (5) perceptions of governance and trust in digital health solutions. These 5 domains served as content frameworks for item construction and were subsequently consolidated into 3 composite scoring domains—self-management, adherence, and governance—based on conceptual clustering and internal consistency analysis (Cronbach α).

#### Instrument Validation

The questionnaire underwent a three-phase validation process: (1) *expert review*—4 specialists in health informatics, oncology, and technology assessed clarity, relevance, and conceptual coherence of each item; (2) *pilot testing*—a focus group of 10 patients and 3 health care professionals evaluated comprehensibility, language adequacy, and logical flow; and (3) *final refinement*—feedback-driven revisions were implemented to eliminate ambiguities and redundancies. All items used closed-ended response formats (single-choice or multiple-choice checkboxes); no open-ended questions were included.

### Data Collection Procedures

Data were collected using the validated questionnaire hosted on the REDCap platform, a secure, Health Insurance Portability and Accountability Act (HIPAA)–compliant web-based application widely used for clinical and academic research. Access was provided via a QR code displayed in strategic institutional locations, including waiting areas of SUS, private oncology outpatient clinics, and physicians’ offices. The questionnaire was self-administered without direct researcher intervention during completion. The expression “in-person” used in previous versions of this manuscript referred exclusively to the recruitment context—face-to-face invitation by research staff—not to the data collection modality, which was entirely digital and self-administered. Participants who experienced difficulty with personal devices were offered an institution-provided tablet. All data were stored in encrypted REDCap servers with access restricted to the principal investigator.

### Data Analysis

All quantitative analyses were conducted using Stata (version 17.0; StataCorp). Descriptive statistics (absolute and relative frequencies, means, and SDs) characterized sample distributions. Independent-samples *t* tests compared composite domain scores between 2-group sociodemographic variables (gender, age group, educational level, income, and self-rated health); 1-way ANOVA was applied for race comparisons across 3 groups, with normality assumed based on the central limit theorem (n>30 per comparison). Pearson correlation coefficients assessed associations among the 3 composite domain scores. A Pearson chi-square goodness-of-fit test examined whether proportions expressing trust differed significantly across the 4 recipient-type categories (health care professionals, hospitals, the pharmaceutical industry, and the government). All tests were 2-tailed; α=.05 was the significance threshold. SEs were estimated under the assumption of a simple random sample, acknowledging the convenience sampling design as a limitation.

Three composite domain scores were calculated by averaging the constituent items (self-management: items 8‐11; adherence: items 12‐16; and governance: items 17‐20), rescaled to 0 to 100. Internal consistency was assessed using Cronbach α, with values ≥0.70 considered acceptable [39].

## Results

### Participant Characteristics

Of 110 patients approached, 104 (94.5%) were included in the final analytic sample (n=6, 5.5% excluded for not providing responses to the composite scoring domain items). Within the 104 included participants, item-level missing data varied by variable (range 1.0%‐7.7%), as reported in Table 1. The sample was predominantly female (63/98, 64.3%) and self-identified as *pardo* (mixed race; 64/98, 65.3%), with a predominance of lower-income participants (55/96, 57.3% earning less than twice the minimum wage).

**Table 1.** Sociodemographic characteristics of the study sample (n=104, with per-variable missing data reported^a^).

Characteristics	Sample, n (%)
Monthly income^b^ (in multiples of the minimum wage; n=96)	
≤Twice	55 (57.3)
>Twice	41 (42.7)
Age group (y; n=98)	
18‐59	53 (54.1)
≥60	45 (45.9)
Sex (n=98)	
Male	35 (35.7)
Female	63 (64.3)
Educational level (n=98)	
High school or higher	46 (46.9)
Elementary school	52 (53.1)
Self-rated health status (n=103)	
Good or very good	58 (56.3)
Fair or poor	45 (43.7)
Race (n=99)	
Black	15 (15.3)
*Pardo* (mixed race)	64 (65.3)
White or Asian	20 (19.4)

a Missing data by variable: monthly income (8/104, 7.7%), age group (6/104, 5.8%), gender (6/104, 5.8%), educational level (6/104, 5.8%), self-rated health status (1/104, 1%), and race (5/104, 5.8%).

b Monthly income is expressed in multiples of the Brazilian minimum wage; in 2023 the monthly minimum wage was BRL 1320 (approximately US $265).

### General Perceptions of Digital Health and Data Sharing

The results indicate high acceptance of digital technologies: 61.4% (68/110) of participants considered the use of technology to integrate health information essential and 86.4% (95/110) were willing to use applications to store health data. Willingness to use prevention-focused applications reached 89.1% (98/110), and 86.4% (95/110) of participants believed that sharing health data is crucial for advances in medical research.

Regarding trust in data sharing, 79.1% felt comfortable sharing information with health care professionals, while trust in hospitals was moderate (57/110, 51.8%) and lower regarding the pharmaceutical industry (17/110, 15.5%). The lowest trust was for sharing with the government ( 11/110, 10%). A Pearson chi-square goodness-of-fit test indicated a statistically significant difference between these proportions (*χ*^2^_3_=210.4; *P*<.001). Guaranteed anonymity and encryption considerably increased comfort in sharing, with 83.6% (92/110) of patients reporting greater willingness under these conditions.

### Analysis of Key Domains and Influence of Sociodemographic Factors

Table 2 presents mean scores for the 3 composite domains—self-management, adherence, and governance—cross-referenced with sociodemographic variables. No statistically significant differences were observed in domain scores based on income, gender, educational level, or self-rated health status (all *P*>.05). Patients aged 18 to 59 years showed significantly higher adherence scores compared with those aged 60 years or older (mean 76.49, SD 19.16 vs mean 65.83, SD 26.66; *t*_95_=2.29; *P*=.02), suggesting a greater affinity for digital solutions among younger participants.

**Table 2.** Relationship between sociodemographic characteristics and domain scores^a^.

Variables	Self-management, mean (SD)	*P* value	Adherence, mean (SD)	*P* value	Governance, mean (SD)	*P* value
Income (in multiples of the monthly minimum wage)		.47		.34		.39
≤Twice	66.36 (14.10)		69.09 (22.17)		60.00 (17.14)	
>Twice	68.75 (18.57)		73.62 (24.27)		63.26 (19.74)	
Age group (years)		.78		.02		.78
18‐59	68.39 (14.31)		76.49 (19.16)		61.85 (19.16)	
≥60	67.44 (18.92)		65.83 (26.66)		60.79 (22.22)	
Gender		.65		.51		.61
Male	66.78 (18.97)		69.45 (25.13)		60.00 (21.78)	
Female	68.36 (14.81)		72.66 (22.09)		61.94 (16.05)	
Educational level		.91		.6		.47
Secondary or higher	67.90 (16.42)		70.24 (23.12)		59.89 (18.17)	
Primary	67.52 (16.54)		72.74 (23.48)		62.58 (18.45)	
Self-rated health		.85		.95		.33
Good or very good	67.52 (16.40)		71.84 (17.88)		59.69 (17.88)	
Fair or poor	68.16 (16.41)		71.55 (22.74)		63.28 (18.61)	
Race		.07		.48		.22
Black	72.50 (14.52)		78.46 (21.40)		63.81 (21.33)	
*Pardo* (mixed-race)	68.94 (15.70)		70.67 (22.59)		62.61 (16.87)	
White or Asian	60.62 (18.03)		70.00 (26.40)		55.00 (19.40)	

a *P* values were obtained using independent-samples *t* tests for 2-group comparisons and 1-way ANOVA for race categories. *t *tests assumed equal variances.

**Figure 2.**
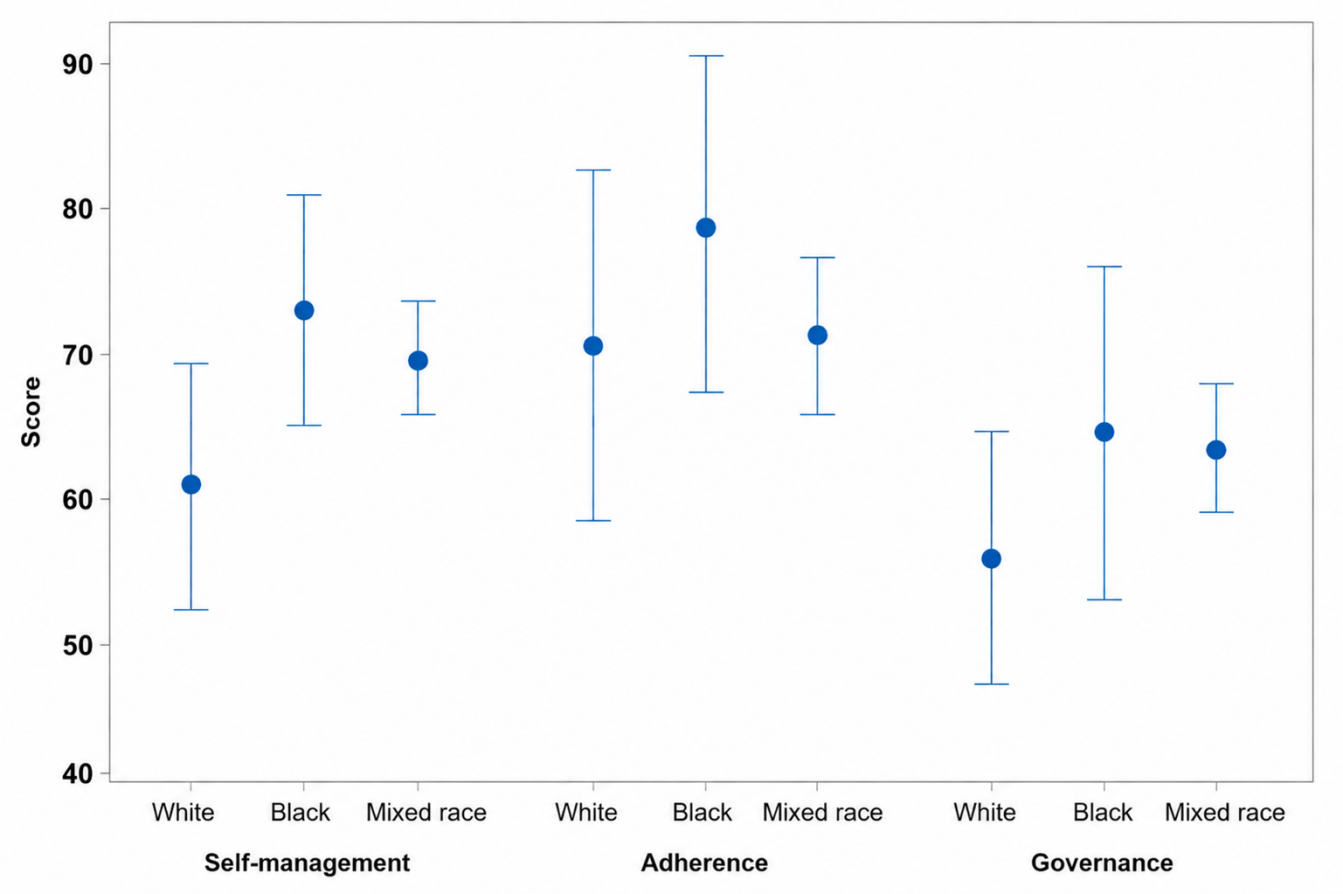
Mean scores and 95% CIs for self-management, adherence, and governance domains, stratified by self-reported race (White, Black, andMixed race). No statistically significant differences were observed between groups (all *P*>.05). Small subgroup sizes (Black: n=15; White: n=20) substantially limit the reliability of between-group estimates.

Race-based comparisons did not reach statistical significance for any domain (all *P*>.05). As shown in Figure 2, Black participants exhibited numerically higher mean scores across all the self-management, adherence, and governance domains; however, these results must be interpreted with considerable caution given the small subgroup sizes (Black: n=15; White: n=20), which substantially limit statistical power and preclude reliable between-group estimates. This is acknowledged as a key limitation (refer to the Limitations section).

### Instrument Reliability

Table 3 presents the mean scores, SDs, and internal consistency (Cronbach α) for each of the 3 composite domains. The adherence domain achieved the highest Cronbach α (α=0.8807), indicating excellent reliability, followed by self-management (α=0.8504) and governance (α=0.7576), the latter still meeting the accepted threshold for health research [39]. The higher mean score for adherence (71.53, SD 23.12) compared with governance (61.25, SD 18.19) suggests that patients felt more personally engaged with digital tools than they trusted institutional actors to manage their data responsibly.

**Table 3.** Mean scores, SDs, and internal consistency (Cronbach α) for the composite domains.

Domain	Scores, mean (SD)		Cronbach α
Self-management	67.80 (16.32)		0.8504
Adherence	71.53 (23.12)		0.8807
Governance	61.25 (18.19)		0.7576

## Discussion

### Principal Findings

This study provides empirical evidence that patients with cancer in Brazil demonstrate high acceptance of digital health technologies and significant willingness to share clinical data, conditional on robust privacy protections and transparent governance. These findings directly address our 3 research questions, confirming the feasibility of patient-centered digital health initiatives within the Brazilian SUS context. As detailed in the Methods, the instrument assessed attitudes toward the functional properties that blockchain enables rather than the technology by name; the findings should therefore be interpreted as reflecting acceptance of blockchain’s underlying value proposition—particularly security and transparency—rather than informed endorsement of the technology itself, a distinction acknowledged in the Limitations section.

### Trust Asymmetry and Implications for Governance

The strong inclination toward digital tool adoption (95/110, 86.4%) is consistent with Brazil’s high smartphone penetration [40-42]. The marked disparity in trust across recipient types—79.1% (87/110) for health care professionals versus 10% (11/110) for the government—reflects a pattern documented in the literature [7,11,43-45]. This asymmetry is explained by 3 interacting factors: the personalized patient–health care provider relationship, institutional distance, and historical privacy scandals that have eroded public trust [6,43,46,47].

These findings have a critical design implication for blockchain systems: the technology’s inherent auditability and immutability may be uniquely positioned to address institutional distrust by allowing patients to monitor who accessed their data, when, and for what purpose [21,29,30]. Technical accountability must be complemented by participatory governance frameworks and regulatory alignment with the LGPD and RNDS standards to achieve social legitimacy [48-50].

Studies indicate that public opinion often associates large institutions with economic interests and decisions disconnected from individual needs [6]. Blockchain-based data-sharing agreements represent essential complementary instruments that translate technical transparency into legally enforceable governance frameworks [51]. Their integration within the RNDS infrastructure—which uses Health Level Seven Fast Healthcare Interoperability Resources (HL7 FHIR) standards and aligns with both LGPD and General Data Protection Regulation principles—provides regulatory scaffolding for ethical blockchain implementation in Brazilian oncology [48,52-54].

### Digital Adoption and Sociodemographic Factors

The significant age-related difference in adherence scores corroborates findings identifying aging as a factor associated with lower digital engagement [55,56]. Lower eHealth literacy among older adults, combined with functional and cognitive changes and reduced social support for technology use, increases hesitation toward digital platforms [56,57]. This reinforces the need for age-adapted digital literacy programs and simplified interface design for older patients with cancer.

The absence of significant differences by income, educational level, and self-rated health status may reflect the relative socioeconomic homogeneity of this single-institution sample (all *P*>.05). However, this does not negate the well-documented role of digital equity in broader populations [58-60]. Digital health programs must proactively incorporate accessibility features and humanized technical support [55].

Race-based differences in domain scores did not reach statistical significance and must be interpreted with particular caution given the small subgroup sizes (Black: n=15; White: n=20), which substantially limit statistical power. The numerical trend of higher scores among Black participants may reflect local context-specific factors—such as community engagement initiatives at Hospital Santa Izabel—rather than generalizable patterns. Future studies with larger, multisite samples should examine racial disparities in digital health acceptance within Brazilian oncology [61,62]. In the absence of adequate statistical power, any interpretation of these results is exploratory and should not be extrapolated. The use of “race” terminology throughout is consistent with Brazilian Institute of Geography and Statistics categorization adopted in the questionnaire.

The analysis of data-sharing conditions highlights that anonymization and encryption substantially increased willingness to share (92/110, 83.6%), providing direct support for blockchain architectures incorporating privacy-preserving cryptographic mechanisms [26,27,30]. When patients retain technical control over who accesses their data, the psychological barrier of institutional distrust is substantially mitigated [29].

The correlation among the 3 domains—self-management, adherence, and governance—suggests that perceived information security acts as a central element in the willingness of patients with cancer to share data in digital environments. Note that these correlations are descriptive associations and do not test formal hypotheses, as the study did not prespecify directional hypotheses; accordingly, we refrain from confirmatory language in their interpretation.

### Limitations

This study has several limitations. First, the convenience sample from a single oncology center in Salvador, Bahia, limits generalizability to the broader, diverse Brazilian cancer population. Probabilistic, multisite sampling is recommended for future studies. Second, the racial subgroup analyses are substantially limited by small cell sizes (Black: n=15; White: n=20), which preclude definitive conclusions. Third, self-selection bias may have inflated acceptance estimates [63]. Fourth, the cross-sectional design precludes assessment of how perceptions evolve over time [64]. Fifth, participants’ prior knowledge of blockchain technology was not formally assessed before questionnaire completion. Therefore, it was not possible to determine the extent to which baseline familiarity with the technology may have influenced perceptions and acceptance. It is plausible that limited blockchain literacy influenced response patterns, such that participants’ acceptance reflected attitudes toward the underlying principles of data governance (eg, security, transparency, and patient control) rather than toward blockchain technology itself. Future studies should assess blockchain literacy at baseline to disentangle knowledge effects from attitudinal responses. Finally, the absence of formal factor analysis to confirm the 3-domain structure is acknowledged; future validation studies should use confirmatory factor analysis with larger samples. In addition, the instrument did not undergo formal cognitive pretesting or debriefing with respondents; although expert review and pilot testing were conducted, cognitive interviewing would have further strengthened content validity and is recommended for future studies. Additionally, SEs were estimated under the assumption of a simple random sample, whereas a convenience sample was used; this assumption may underestimate variance and further limit the generalizability of the results.

### Future Directions

Future research should incorporate probabilistic, multiregion sampling and longitudinal designs to track the evolution of patient trust and digital acceptance. Mixed methods studies integrating in-depth interviews and focus groups would provide deeper insight into contextual factors shaping data-sharing decisions. Intervention studies evaluating real-world blockchain-based platforms—with patient-controlled consent mechanisms and transparent audit logs—should assess their impact on trust and clinical outcomes. Studies should formally assess blockchain literacy at baseline. Targeted digital inclusion programs for older adults and socioeconomically disadvantaged patients are essential to ensure equitable access to health innovations.

### Conclusions

This study demonstrates that patients with cancer in Brazil exhibit high acceptance of digital health technologies and significant openness to clinical data sharing, provided that strong privacy protections, transparent governance, and patient-centered control are guaranteed. Critically, patient support for the proposed blockchain-based framework reflects acceptance of its functional principles—security, anonymization, and auditability—rather than informed familiarity with blockchain as a technology. This distinction has direct implications for future implementation strategies: educational components addressing blockchain literacy should be integral to any deployment. The marked trust asymmetry across recipient types underscores that technological innovation alone is insufficient. When implemented with adherence to LGPD regulations and integration with existing health infrastructure (RNDS and Conecte SUS), blockchain-based health data management systems represent a feasible and socially acceptable approach to transforming oncology data governance in Brazil.

## Supplementary material

10.2196/89278 Multimedia Appendix 1 Institutional review board approval document (consubstantiated opinion) issued by the research ethics committee (Comitê de Ética em Pesquisa) of Hospital Santa Izabel. The document certifies the ethics approval of the study under Certificate of Presentation for Ethical Consideration number 70726523.3.0000.5520 and approval opinion number 068562/2023, in accordance with national ethical and regulatory standards for research involving human participants.

10.2196/89278 Multimedia Appendix 2 Research questionnaire (English translation; the instrument was administered in Brazilian Portuguese).

10.2196/89278 Checklist 1 STROBE checklist for cross-sectional studies, completed by the authors.

## Abbreviations

ECOG: Eastern Cooperative Oncology Group
EHR: electronic health record
HIPAA: Health Insurance Portability and Accountability Act
HL7 FHIR: Health Level Seven Fast Healthcare Interoperability Resources
LGPD: Lei Geral de Proteção de Dados
REDCap: Research Electronic Data Capture
RNDS: Rede Nacional de Dados em Saúde
STROBE: Strengthening the Reporting of Observational Studies in Epidemiology
SUS: Sistema Único de Saúde
