# Supplementary figures and images for: Patient Perceptions and Acceptance of Blockchain-Based Health Data Sharing in Oncology: Cross-Sectional Survey
Source: JMIR Form Res. 2026 Jun 25;10:e89278. doi: 10.2196/89278 (PMC13295423; doi:10.2196/89278)

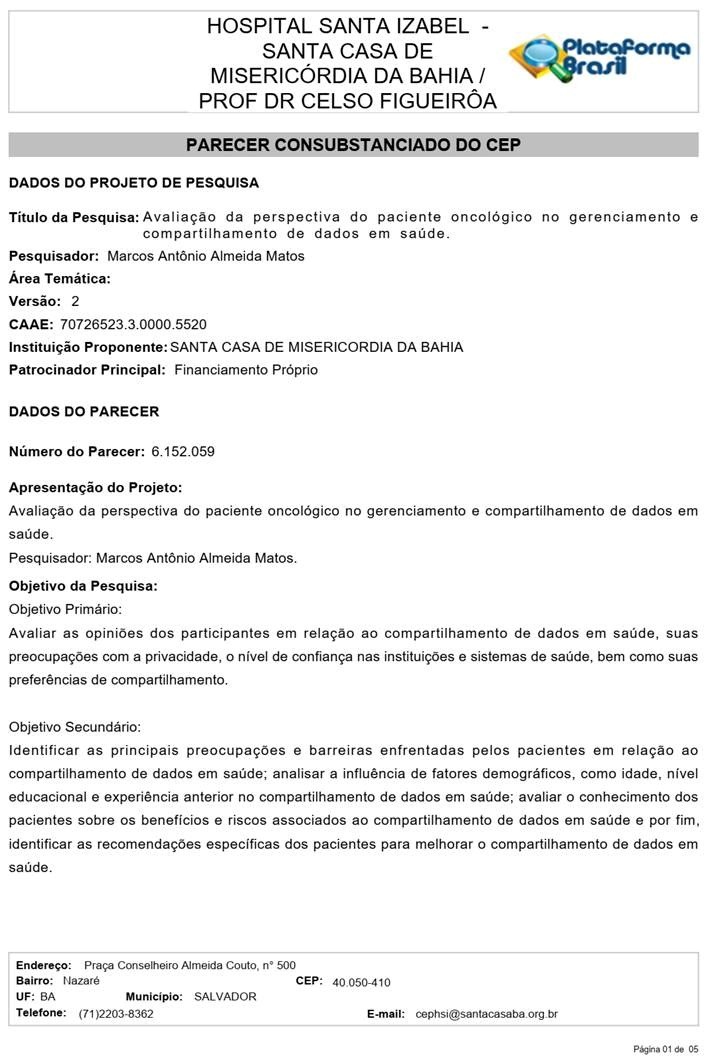


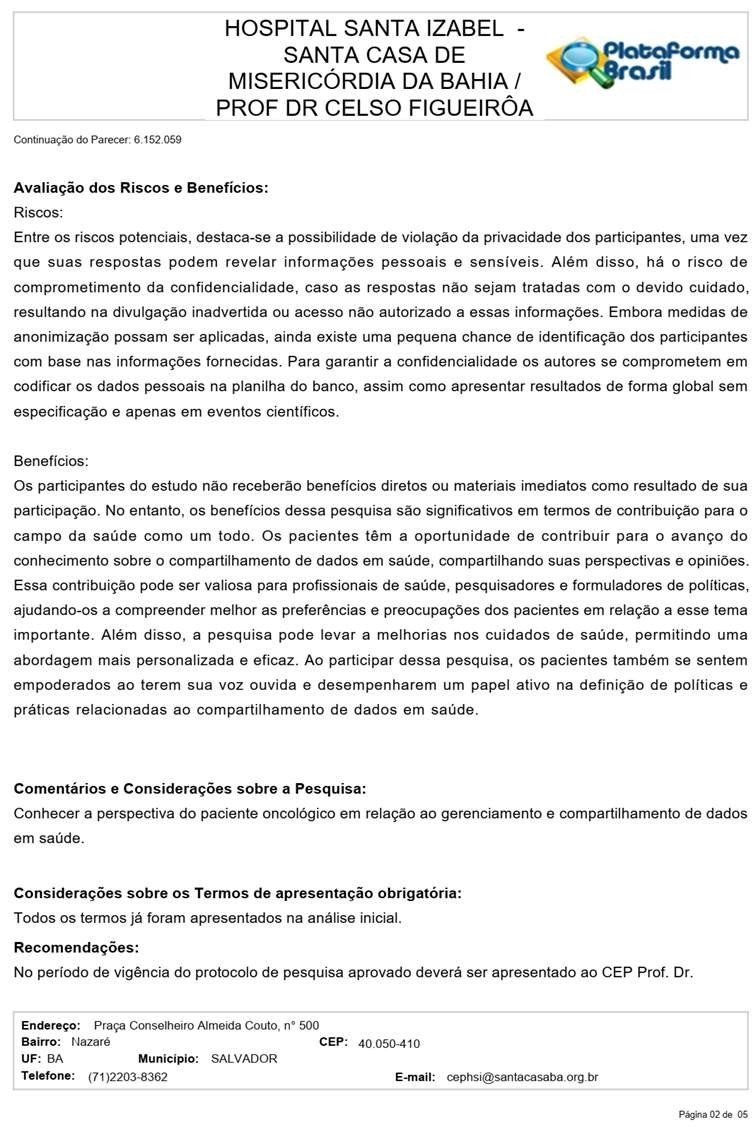


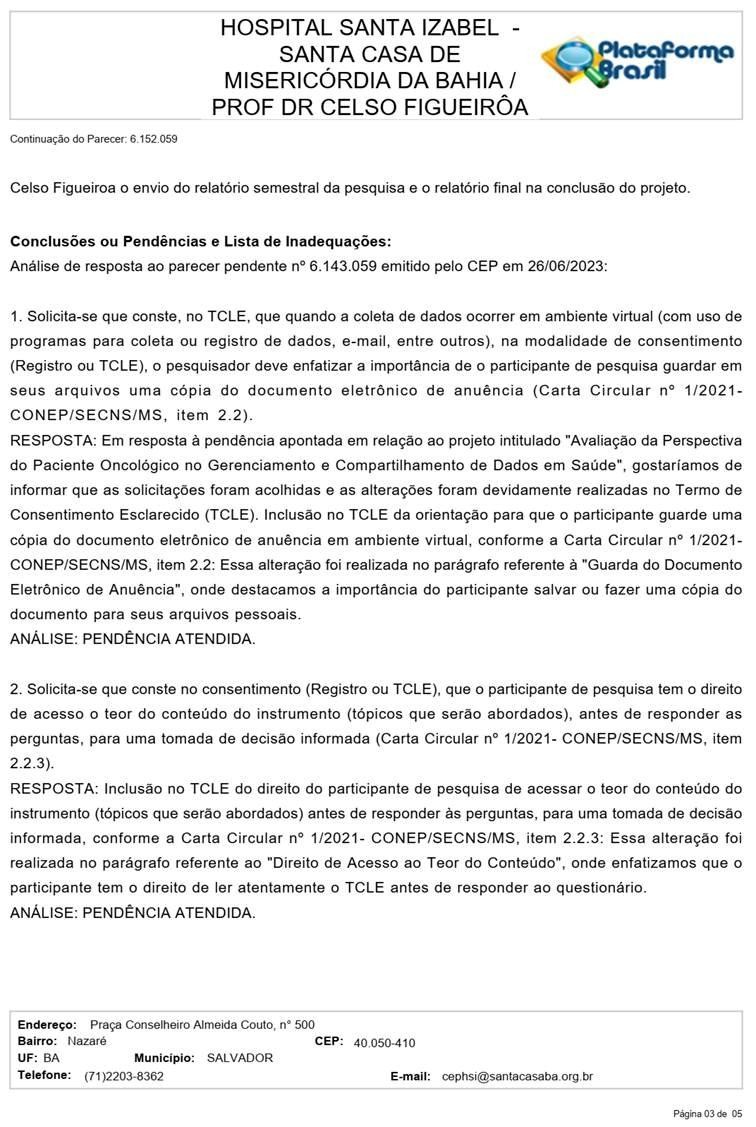


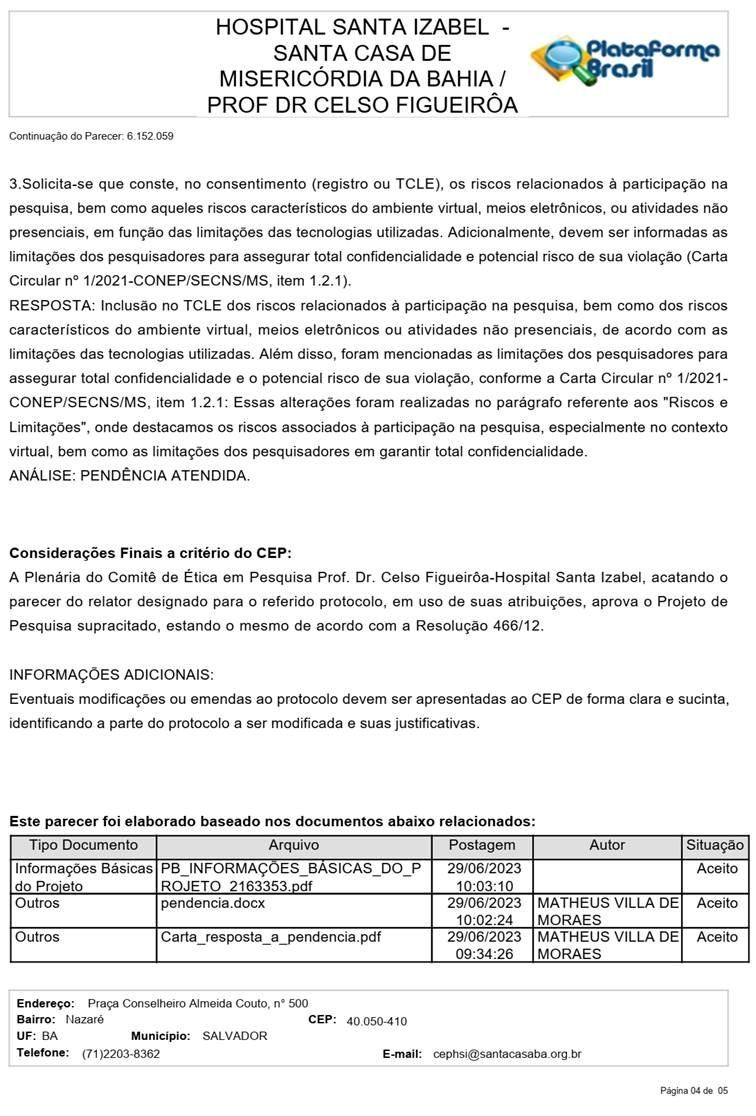


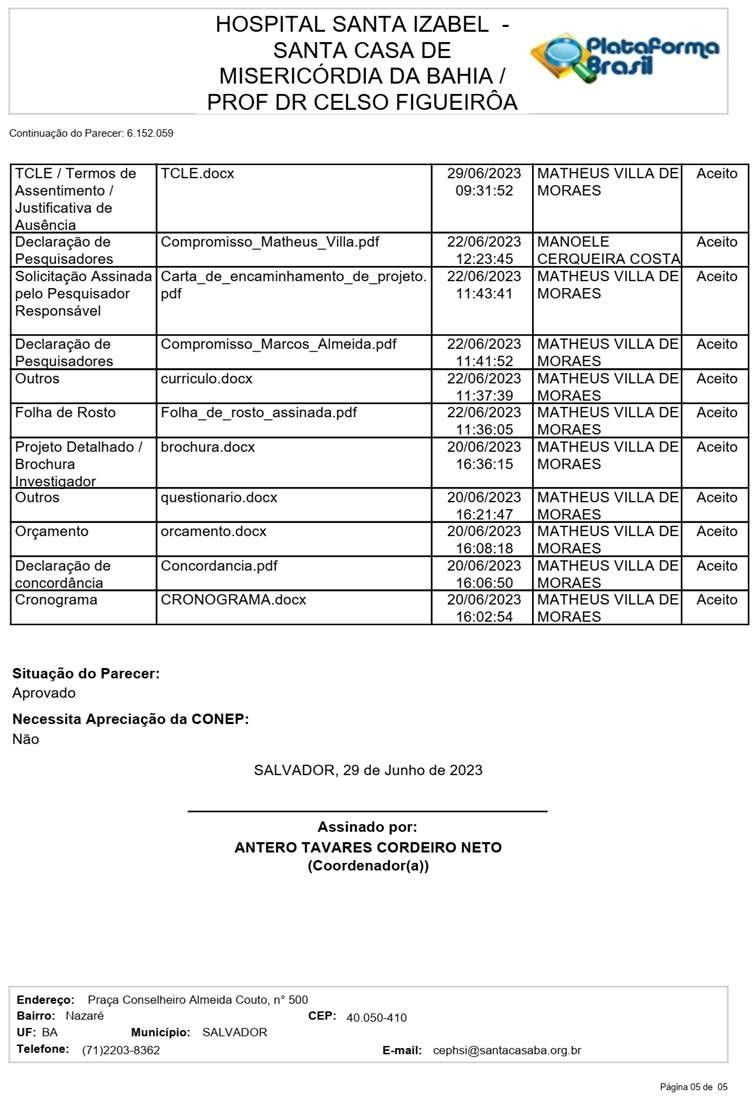


.

Supplement: Multimedia Appendix 1 [file formative-v10-e89278-s001.docx]
