# Supplementary material for: Patient Perceptions and Acceptance of Blockchain-Based Health Data Sharing in Oncology: Cross-Sectional Survey
Source: JMIR Form Res. 2026 Jun 25;10:e89278. doi: 10.2196/89278 (PMC13295423; doi:10.2196/89278)
Supplement: Multimedia Appendix 2 [file formative-v10-e89278-s002.docx]

**1. What is your age? (Enter your age in years)**

**2. What is your gender?**

( ) Male

( ) Female

**3. How do you identify in terms of color/race?**

( ) White

( ) Black

( ) Brown (Pardo)

( ) Yellow (Asian descent)

( ) Indigenous

( ) Not declared

**4. What is your level of education?**

( ) Complete primary education

( ) Incomplete primary education

( ) Complete secondary education

( ) Incomplete secondary education

( ) Higher education

**5. What is your monthly income?**

( ) Up to 2 minimum wages

( ) From 2 to 4 minimum wages

( ) From 4 to 7 minimum wages

( ) More than 7 minimum wages

**6. How do you rate your overall health status?**

( ) Very good

( ) Good

( ) Fair

( ) Poor

( ) Very poor

**7. Which actions do you consider important to improve health management in the country?**

*(Select as many as you consider necessary)*

[ ] Technology to integrate health information

[ ] Universal health record

[ ] Health monitoring

[ ] Faster processes

[ ] Security and privacy

**8. Would you be willing to use a mobile phone application where it would be possible to store data on your health information, medications, and exams?**

( ) Yes

( ) I don’t know

( ) No

**9. Would you like all your medical information from different visits and healthcare providers to be stored in a database automatically?**

( ) Yes

( ) No

( ) I don’t know

**10. Would you share your health data among the healthcare institutions you attend for the purposes of medical research and monitoring of your health?**

( ) Yes

( ) No

( ) I don’t know

**11. With whom would you feel comfortable sharing your health data?**

*(Select as many as you consider necessary)*

[ ] Healthcare professionals

[ ] Hospitals

[ ] Health insurance plans

[ ] Government

[ ] Pharmaceutical industry

**12. In which situations would you share your health data?**

*(Select as many as you consider necessary)*

[ ] Improvement of my health care

[ ] Contributing to medical research

[ ] Helping to develop new medications or treatments

[ ] Speeding up processes

[ ] Security and privacy of my information

**13. Would you feel more comfortable sharing your health data if it were guaranteed that your personal information would be anonymous and protected by encryption (the use of a code to ensure information secrecy and confidentiality)?**

( ) Yes

( ) No

( ) I don’t know

**14. Do you believe that healthcare institutions, research bodies, and pharmaceutical companies have adequate policies to protect patient health data?**

( ) Yes

( ) No

( ) I don’t know

**15. Do you believe that sharing health data is important for advances in medical research and the development of new treatments?**

( ) Yes

( ) No

( ) I don’t know

**16. Would you share your health data among institutions and healthcare professionals through a QR code ("key, password"), so that this information would be organized in a clear and objective way? (willingness to share + security)**

( ) Yes

( ) No

( ) I don’t know

**17. Would you use a health application that would assist in disease prevention and health promotion, disease control, surveillance, and monitoring of your health?**

( ) Yes

( ) No

( ) I don’t know

**18. Which of the following situations do you consider most appropriate for releasing your health data for research purposes?**

*(Select as many as you consider necessary)*

[ ] At the request of my doctor or healthcare professional

[ ] When requested by researchers

[ ] When requested by the authorities

[ ] At any time and place using a mobile application

**19. Which information do you consider important regarding legislation for health data sharing?**

*(Select as many as you consider necessary)*

[ ] Concerns regarding data breaches

[ ] Confidentiality

[ ] Security

[ ] Structuring the information related to care/visits

[ ] Open/free applications or with no usage costs

**20. Which items do you consider important in a health application?**

*(Select as many as you consider necessary)*

[ ] Names of medications in use

[ ] Adequate health monitoring

[ ] History of exams and treatment

[ ] Medical alert

[ ] Allergy

[ ] Blood type

***Note.*** *Survey instrument used to assess oncology patients’ perceptions of digital health, data sharing, privacy, governance, and acceptance of blockchain-based solutions. The questionnaire comprises 20 items covering sociodemographic characteristics, attitudes toward digital technologies, willingness to share health data, perceptions of privacy and security, and expectations regarding governance and legislation related to health data use. All items used closed-ended response formats (single-choice or multiple-choice checkboxes); no open-ended questions were included. The questionnaire was developed and administered entirely in Brazilian Portuguese; this English version is provided for reviewer reference only.*
