# Supplementary material for: Patient Perceptions and Acceptance of Blockchain-Based Health Data Sharing in Oncology: Cross-Sectional Survey
Source: JMIR Form Res. 2026 Jun 25;10:e89278. doi: 10.2196/89278 (PMC13295423; doi:10.2196/89278)
Supplement: Checklist 1 [file formative-v10-e89278-s003.docx]

| **Item No** | **Recommendation** | **Reported?** | **Location in manuscript / Explanation** |
| --- | --- | --- | --- |
| **TITLE AND ABSTRACT** | | | |
| **1a** | (a) Indicate the study's design with a commonly used term in the title or the abstract | **Yes** | Title: "...A Cross-Sectional Survey of Patient Perceptions and Acceptance in Brazil." Abstract, Methods section: "An exploratory, descriptive, cross-sectional self-report survey was conducted..." |
| **1b** | (b) Provide in the abstract an informative and balanced summary of what was done and what was found | **Yes** | Abstract (444 words): structured in five components (Background, Objective, Methods, Results, Conclusions). Includes setting, sample, instrument, outcomes, key statistics (chi-squared=210.4, P<.001; P=.024; Cronbach's alpha values), and conclusions. |
| **INTRODUCTION** | | | |
| **2** | Explain the scientific background and rationale for the investigation being reported | **Yes** | Introduction (paragraphs 1–6): Covers fragmentation of EHR systems in oncology, limitations of current health data governance in Brazil (SUS, RNDS), blockchain as a proposed solution, and the gap in patient-centered evidence regarding digital health data sharing. |
| **3** | State specific objectives, including any prespecified hypotheses | **Yes** | Introduction, final paragraph: Three explicit research questions stated: (1) level of digital health tool acceptance; (2) sociodemographic factors associated with willingness to share data; (3) feasibility and acceptability of a blockchain-based approach. No formal directional hypotheses were prespecified (exploratory study). |
| **METHODS** | | | |
| **4** | Present key elements of study design early in the paper | **Yes** | Methods, Study Design section: "This exploratory and descriptive study adopted a quantitative cross-sectional self-report survey design." Design stated in Abstract and Introduction as well. |
| **5** | Describe the setting, locations, and relevant dates, including periods of recruitment, exposure, follow-up, and data collection | **Yes** | Methods, Study Setting and Recruitment: Hospital Santa Izabel, Salvador, Bahia, Brazil — national oncology reference center integrated into the SUS ecosystem. Data collection: September–November 2023. No follow-up (cross-sectional design). |
| **6a** | (a) Give the eligibility criteria, and the sources and methods of selection of participants | **Yes** | Methods, Participants: Inclusion criteria: adults ≥18 years, undergoing active oncology treatment or follow-up, ECOG 0–2. Exclusion: ECOG 3–4; did not provide responses to the items comprising the three composite scoring domains. Systematic convenience sampling during outpatient appointments at Hospital Santa Izabel. |
| **7** | Clearly define all outcomes, exposures, predictors, potential confounders, and effect modifiers | **Yes** | Methods, Questionnaire Development and Validation: Three composite domain scores defined (Self-Management: items 8–11; Adherence: items 12–16; Governance: items 17–20). Sociodemographic variables (income, age, gender, race, education, self-rated health) serve as predictors. No confounders adjusted for (descriptive/exploratory study). |
| **8** | For each variable of interest, give sources of data and details of methods of assessment | **Yes** | Methods, Questionnaire Development: 20-item instrument developed de novo, validated via expert panel (n=4 specialists) and pilot testing (n=10 patients, n=3 professionals). Hosted on REDCap. Data Collection Procedures: QR-code access, self-administered. All items closed-ended (single- or multiple-choice). |
| **9** | Describe any efforts to address potential sources of bias | **Yes** | Methods, Data Collection Procedures: self-administered without researcher intervention to minimize social desirability bias. Limitations section: self-selection bias acknowledged (participants with greater digital familiarity may have been more likely to respond); single-center design limits generalizability. |
| **10** | Explain how the study size was arrived at | **Yes** | Methods, Participants: Sample of n≥100 sufficient to detect medium-sized effects (Cohen's f=0.25) with ~80% power in ANOVA comparisons between 2–4 groups (Terwee et al., 2007). Determined by institutional feasibility; convenience sample acknowledged as limitation. |
| **11** | Explain how quantitative variables were handled in the analyses | **Yes** | Methods, Data Analysis: Continuous age grouped into two categories (18–59 years; ≥60 years) for comparative analyses. Domain scores averaged from constituent items and rescaled to 0–100. Income categorized as ≤2 vs. >2 minimum wages. Groupings justified by clinical and methodological rationale. |
| **12a** | (a) Describe all statistical methods, including those used to control for confounding | **Yes** | Methods, Data Analysis: Stata 17.0. Descriptive statistics (frequencies, means, SD). Independent t-tests for two-group comparisons; one-way ANOVA for race (three groups). Pearson correlations for inter-domain associations. Pearson chi-square goodness-of-fit test for trust proportions. Exploratory study — no confounding adjustment performed. |
| **12b** | (b) Describe any methods used to examine subgroups and interactions | **Yes** | Methods, Data Analysis / Results, Table 2: Subgroup analyses conducted for all six sociodemographic variables (income, age group, gender, educational level, self-rated health, race) across the three composite domain scores. No interaction analyses (exploratory design). |
| **12c** | (c) Explain how missing data were addressed | **Yes** | Methods, Participants: six participants excluded for not responding to composite scoring domain items. Results, Table 1: per-variable missing data reported (range: 1.0%–7.7%); participants with missing data on a specific variable excluded from that variable's analysis only (complete-case analysis per variable). |
| **12d** | (d) Describe analytical methods taking account of sampling strategy | **Yes** | Methods, Data Analysis: Standard errors estimated under the assumption of a simple random sample. Convenience sampling design acknowledged as a limitation affecting generalizability; noted explicitly in the Limitations section. |
| **12e** | (e) Describe any sensitivity analyses | **N/A** | No sensitivity analyses were conducted given the exploratory, descriptive nature of the study. This is noted in the Limitations section. |
| **RESULTS** | | | |
| **13a** | (a) Report numbers of individuals at each stage of study | **Yes** | Results, Participant Characteristics: 110 patients approached; 104 included in final analytic sample; 6 excluded (did not respond to composite scoring domain items). Response rate: 94.5%. |
| **13b** | (b) Give reasons for non-participation at each stage | **Yes** | Results, Participant Characteristics: The 6 excluded participants did not provide responses to the items comprising the three composite scoring domains (Self-Management, Adherence, Governance). No flow diagram provided (single-stage recruitment without eligibility screening data). |
| **13c** | (c) Consider use of a flow diagram | **N/A** | Single-stage recruitment (outpatient visits); no multi-stage screening. Participant flow described in narrative in Results section. A flow diagram is not applicable given the simple sampling structure. |
| **14a** | (a) Give characteristics of study participants | **Yes** | Results, Table 1: Full sociodemographic profile reported (income, age group, gender, educational level, self-rated health, race) with frequencies and percentages for all categories. |
| **14b** | (b) Indicate number of participants with missing data for each variable of interest | **Yes** | Results, Table 1: Missing data column included for each variable (range: 1.0%–7.7%). Footnote explains that participants with missing data on a specific variable were excluded only from that variable's analysis. |
| **15** | Report numbers of outcome events or summary measures | **Yes** | Results, Table 3: Mean scores and standard deviations reported for all three composite domains (Self-Management: 67.80±16.32; Adherence: 71.53±23.12; Governance: 61.25±18.19). Cronbach's alpha reported for each domain. |
| **16a** | (a) Give unadjusted estimates and their precision | **Yes** | Results, Table 2: Mean ± SD reported for all domain scores by sociodemographic group. P values from t-tests and one-way ANOVA reported for all comparisons. No confounder-adjusted estimates (exploratory descriptive study). |
| **16b** | (b) Report category boundaries when continuous variables were categorized | **Yes** | Methods, Data Analysis / Results: Age categorized as 18–59 years and ≥60 years; boundaries reported in Table 1 and throughout the text. Income: ≤2 minimum wages vs. >2 minimum wages; boundaries defined and reported. |
| **16c** | (c) If relevant, consider translating estimates of relative risk into absolute risk | **N/A** | No relative risk estimates calculated. Study reports mean domain scores and proportions (descriptive/cross-sectional design without incidence or risk estimation). |
| **17** | Report other analyses done — e.g. analyses of subgroups and interactions, and sensitivity analyses | **Yes** | Results, Analysis of Key Domains: Subgroup analyses across six sociodemographic variables × three domain scores reported in Table 2 with full test statistics. Pearson correlations between domain scores reported in text. Race-based comparisons explicitly caveated due to small subgroup sizes (Black: n=15; White/Yellow: n=19). |
| **DISCUSSION** | | | |
| **18** | Summarise key results with reference to study objectives | **Yes** | Discussion, Principal Findings: Key results summarised in relation to the three research questions: (1) high digital health tool acceptance (86.4% willing to use apps); (2) significant age-group difference in Adherence scores (P=.024); (3) trust asymmetry across recipient types (chi-squared=210.4, P<.001) supporting blockchain feasibility. |
| **19** | Discuss limitations, taking into account sources of potential bias or imprecision | **Yes** | Discussion, Limitations: Six limitations discussed: (1) convenience sample from single center — limits generalizability; (2) small racial subgroup sizes — limits between-group comparisons; (3) self-selection bias — may inflate acceptance estimates; (4) cross-sectional design — precludes temporal assessment; (5) blockchain literacy not formally assessed — may have influenced responses; (6) absence of formal factor analysis. |
| **20** | Give a cautious overall interpretation of results considering objectives, limitations, and other evidence | **Yes** | Discussion, subsections and Conclusions: Results interpreted with hedged language ("suggests," "is consistent with," "supports the interpretation that"). Findings situated in relation to prior literature (Kalkman et al., 2022; Grande et al., 2015; Tian et al., 2019). Patient support explicitly described as acceptance of blockchain's functional principles rather than the technology itself. |
| **21** | Discuss the generalisability (external validity) of the study results | **Yes** | Discussion, Limitations and Future Directions: External validity limitations acknowledged — single-center in Salvador, Bahia; does not capture Brazil's ethnic, socioeconomic, and geographic diversity. Probabilistic, multi-region sampling recommended for future studies. |
| **OTHER INFORMATION** | | | |
| **22** | Give the source of funding and the role of the funders for the present study | **Yes** | Funding section: "This research did not receive any specific grant from funding agencies in the public, commercial, or not-for-profit sectors." No funder role to declare. |

**Legend:** Yes = item reported in the manuscript; N/A = item not applicable to this study design. *No items were assessed as "Not reported."*

**Reference:** von Elm E, Altman DG, Egger M, Pocock SJ, Gøtzsche PC, Vandenbroucke JP; STROBE Initiative. The Strengthening the Reporting of Observational Studies in Epidemiology (STROBE) statement: guidelines for reporting observational studies. Lancet. 2007;370(9596):1453-1457. doi:10.1016/S0140-6736(07)61602-X
